# Author Correction: Isolation of infectious Lloviu virus from Schreiber’s bats in Hungary

**DOI:** 10.1038/s41467-022-32735-w

**Published:** 2022-09-06

**Authors:** Gábor Kemenesi, Gábor E. Tóth, Martin Mayora-Neto, Simon Scott, Nigel Temperton, Edward Wright, Elke Mühlberger, Adam J. Hume, Ellen L. Suder, Brigitta Zana, Sándor A. Boldogh, Tamás Görföl, Péter Estók, Tamara Szentiványi, Zsófia Lanszki, Balázs A. Somogyi, Ágnes Nagy, Csaba I. Pereszlényi, Gábor Dudás, Fanni Földes, Kornélia Kurucz, Mónika Madai, Safia Zeghbib, Piet Maes, Bert Vanmechelen, Ferenc Jakab

**Affiliations:** 1 National Laboratory of Virology, Szentágothai Research Centre, University of Pécs, Pécs, Hungary; 2 Institute of Biology, Faculty of Sciences, University of Pécs, Pécs, Hungary; 3 Viral Pseudotype Unit, Medway School of Pharmacy, Chatham Maritime, Universities of Kent & Greenwich, Kent, UK; 4 Viral Pseudotype Unit, School of Life Sciences, University of Sussex, Falmer, Sussex, UK; 5 Department of Microbiology, Boston University School of Medicine, Boston, MA USA; 6 Aggtelek National Park Directorate, Jósvafő, Hungary; 7 Department of Zoology, Eszterházy Károly University, Eger, Hungary; 8 Institute of Ecology and Botany, ÖK Centre for Ecological Research, Vácrátót, Hungary; 9 Medical Centre, Hungarian Defence Forces, Budapest, Hungary; 10 Leuven, Rega Institute, Department of Microbiology, Immunology and Transplantation, Laboratory of Clinical and Epidemiological Virology, Leuven, Belgium

**Keywords:** Viral reservoirs, Pathogens, Virus-host interactions

Correction to: *Nature Communications* 10.1038/s41467-022-29298-1, published online 31 March 2022

The original version of this Article omitted from the author list the 14th author Tamara Szentiványi, who is from the ‘Institute of Ecology and Botany, ÖK Centre for Ecological Research, Vácrátót, Hungary’. Consequently, ‘T.S.’ was added to the Author Contributions: ‘P.E., T.S. ectoparasite identification.’ This has been corrected in both the PDF and HTML versions of the Article.

