# Supplementary material for: Isolation of infectious Lloviu virus from Schreiber’s bats in Hungary
Source: Nat Commun. 2022 Mar 31;13:1706. doi: 10.1038/s41467-022-29298-1 (PMC8971391; doi:10.1038/s41467-022-29298-1)
Supplement: Supplementary file 1 — Supplementary Information [file 41467_2022_29298_MOESM1_ESM.pdf]

Supplementary Information

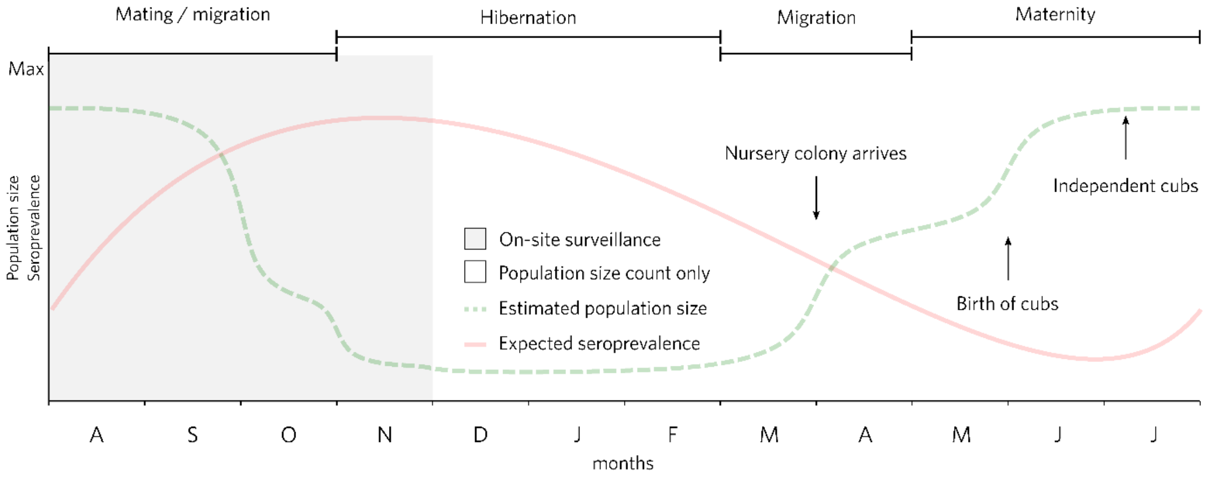

Supplementary Fig. 1. Annual cycle of the Schreiber's bats colony at the investigated mine in Northeast Hungary. During hibernation, regular observation was performed of the bat colony with no active sampling. Grey background represents the timeframe of active bat sampling and sample collection. Population size fluctuation is based on colony size monitoring data in recent years.

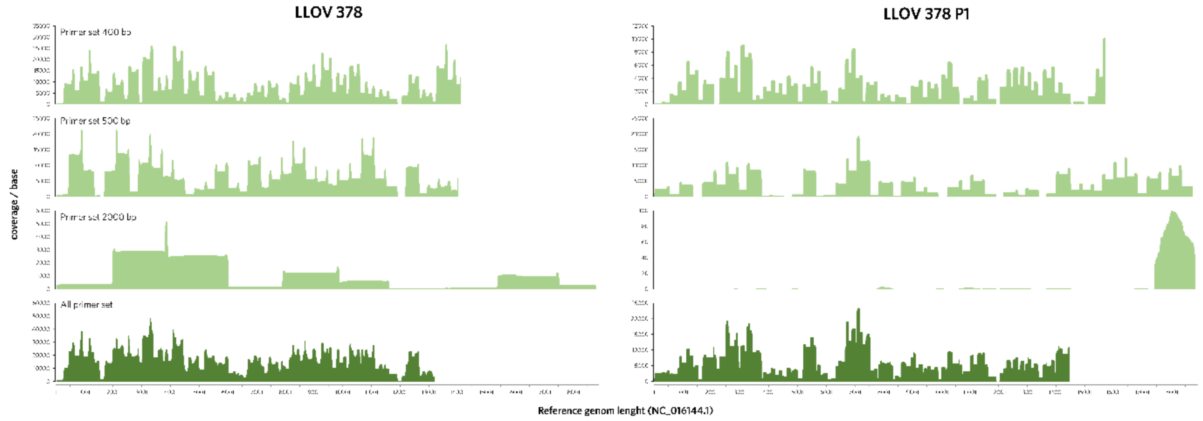

LLOV 378			
	Mean coverage	Number of low coverage sites (< 30)	Number of low coverage sites (< 50)
**Primer set 400**	9208	236	239
**Primer set 500**	7135	60	143
**Primer set 2000**	1001	29	30
**All primer set**	17345	29	28

LLOV 378 P1			
	Mean coverage	Number of low coverage sites (< 30)	Number of low coverage sites (< 50)
**Primer set 400**	2716	352	603
**Primer set 500**	3825	1436	1440
**Primer set 2000**	6	17410	17597
**All primer set**	6547	115	117

Supplementary Fig. 2. Sequense read coverage of sequenced samples LLOV_378 and LLOV:378_P1 alongside the reference LLOV genome sequence (NC_016144). Main characteristics of coverage (mean, extent) with the three different primer sets are shown in the tables below the plots.

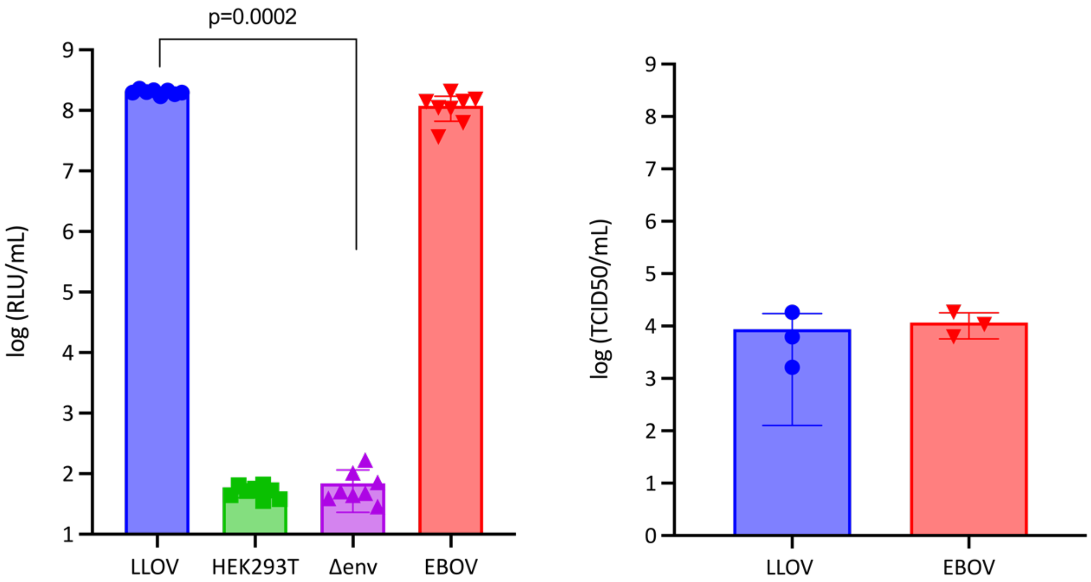

Supplementary Fig. 3. Lentiviral LLOV PV titres. An EBOV GP PV generated and titrated previously was included as a positive control in – infectivity and Panel B – $TCID_{50}$ assays. Uninfected cells (HEK293T) and particles devoid of GP (Δenv) were included as negative controls. Average titres three independent experiments shown (n=3). Error bars indicate mean ± SD. Statistical significance (p=0.0002 Mann-Whitney Two-tailed test) in comparison to Δenv determined with Prism 8 software. Source data are provided as a Source Data file.

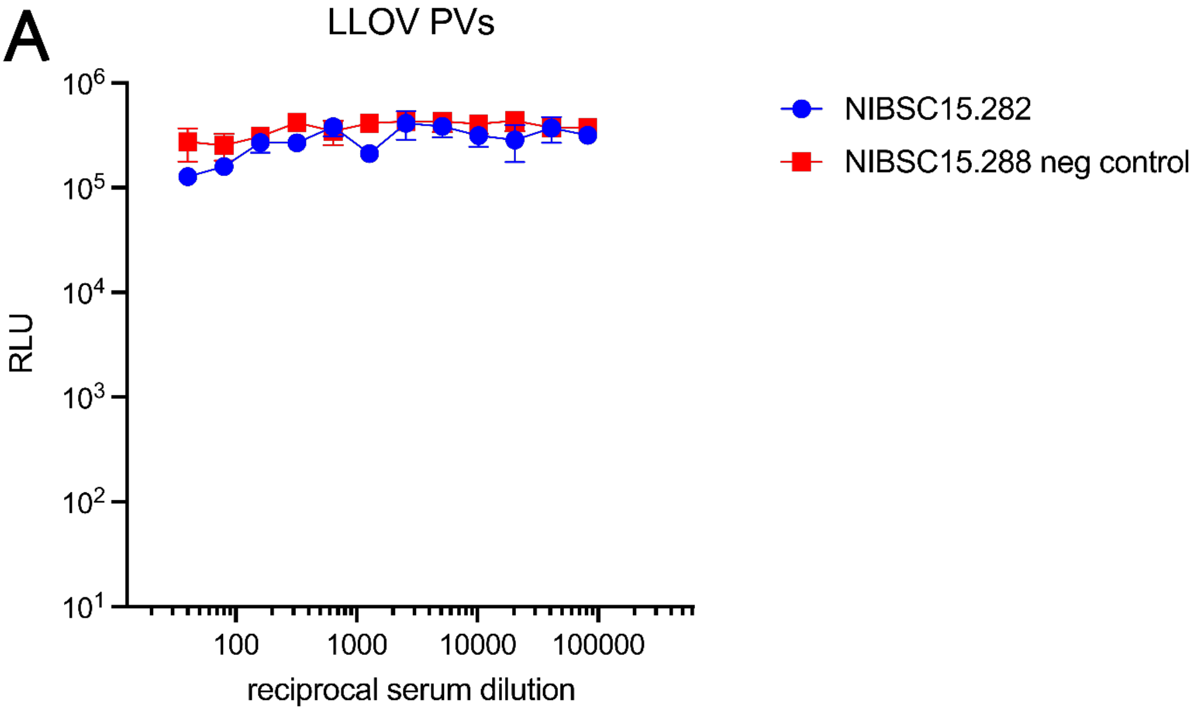

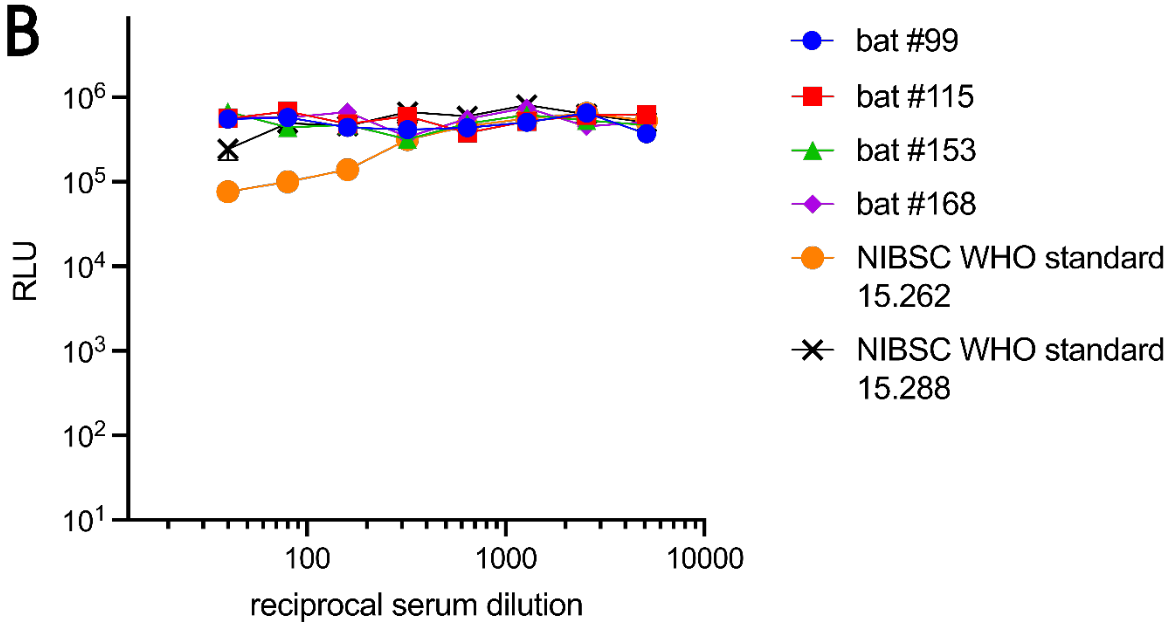

Supplementary Fig. 4. Cross-reactivity tests. Panel A: Test of WHO standard sera on the LLOV PV system. One experiment was performed with three technical replicates. Error bars indicate mean ± SD. Panel B: LLOV seropositive (#99 and #115) and seronegative (#153 and #168) bat sera in the Ebola virus PVNT assay. Controls were WHO standards from NIBSC. RLU refers to relative light unit. Single measurements were performed except for the standard (n=2). Error bars indicate mean ± SD. Source data are provided as a Source Data file.

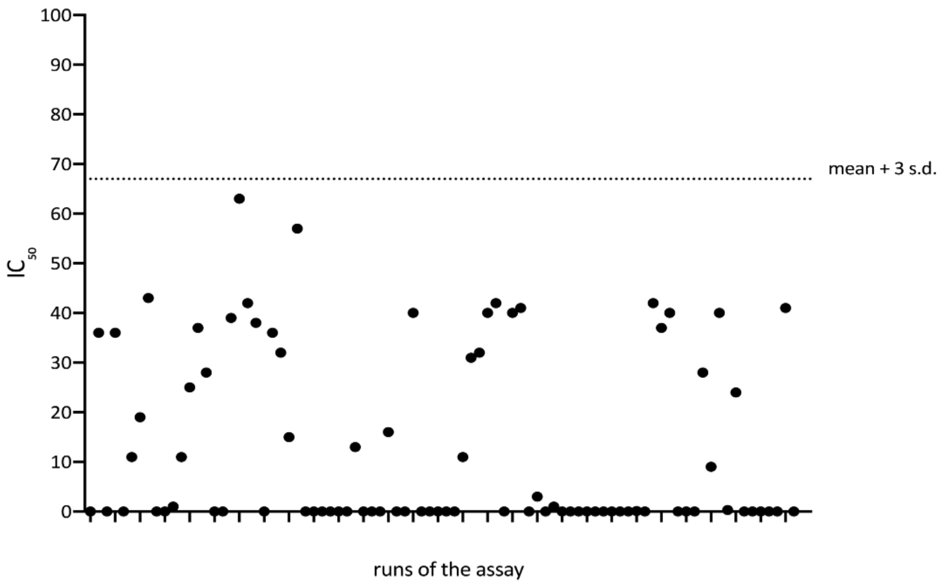

Supplementary Fig. 5. PVNT cut-off determination for positive samples. Samples included showed no significant (antibody mediated) reduction in luminescence compared to PV only samples (no serum). 46 tests did not yield an IC_{50}. The remaining 40 tests were found to have a low range of IC_{50} titres, but showed no significant reduction in luminescence and therefore were included in the analysis. Mean, standard deviation (s.d.) and cut-off point calculated with Prism 8.

Supplementary Table 1. In vitro infection of Lloviu virus on monkey and human cell lines. Cells were infected with MOI 0.01 of the virus isolate. DPI refers to days post-virus infection.

	**VeroE6**	**SH-SY5Y**	**HepG2**	**HCC78**	**HCT116**
	Monkey kidney cells	Human neuroblastoma	Human hepatocyte carcinoma	Human lung adenocarcinoma	Human colon carcinoma
0 DPI Ct value	24.03	27.33	24.99	26.15	27.47
10 DPI supernatant Ct value	21.5	24.36	22.55	23.11	24.87
