# Supplementary material for: Isolation of infectious Lloviu virus from Schreiber’s bats in Hungary
Source: Nat Commun. 2022 Mar 31;13:1706. doi: 10.1038/s41467-022-29298-1 (PMC8971391; doi:10.1038/s41467-022-29298-1)
Supplement: Supplementary file 3 — Description of Additional Supplementary Files [file 41467_2022_29298_MOESM3_ESM.pdf]

Description of Additional Supplementary Files

File Name: Supplementary Data 1
Description: The dataset contains the processed samples and measurements that are involved in this study. There are four sheets (Live_animals, Dead_animals, Ectoparasites, Live animals + ectoparasites) with all available details (species, collection date, sex) about the collected specimens and the result of LLOV detection or serological status. Abbreviations: F – female, M – Male, neg – negative, POS – positive, NA - not applicable, NT – not tested

File Name: Supplementary Movie 1
Description: CytoSMART live cell imaging system video of the progress of cytopathic effect on SuBK12-08 cell line during LLOV infection.

File Name: Supplementary Movie 2
Description: CytoSMART live cell imaging system video of the progress of cytopathic effect on Vero E6 cell line during LLOV infection.
