# Supplementary material for: Isolation of infectious Lloviu virus from Schreiber’s bats in Hungary
Source: Nat Commun. 2022 Mar 31;13:1706. doi: 10.1038/s41467-022-29298-1 (PMC8971391; doi:10.1038/s41467-022-29298-1)
Supplement: Supplementary file 7 — Reporting Summary [file 41467_2022_29298_MOESM7_ESM.pdf]

nature portfolio

Corresponding author(s): Gábor Kemenesi

Last updated by author(s): Feb 19, 2022

Reporting Summary

Nature Portfolio wishes to improve the reproducibility of the work that we publish. This form provides structure for consistency and transparency in reporting. For further information on Nature Portfolio policies, see our Editorial Policies and the Editorial Policy Checklist.

Statistics

For all statistical analyses, confirm that the following items are present in the figure legend, table legend, main text, or Methods section.

n/a	Confirmed	
☐	☒	The exact sample size (*n*) for each experimental group/condition, given as a discrete number and unit of measurement
☐	☒	A statement on whether measurements were taken from distinct samples or whether the same sample was measured repeatedly
☐	☒	The statistical test(s) used AND whether they are one- or two-sided<br>*Only common tests should be described solely by name; describe more complex techniques in the Methods section.*
☒	☐	A description of all covariates tested
☒	☐	A description of any assumptions or corrections, such as tests of normality and adjustment for multiple comparisons
☐	☒	A full description of the statistical parameters including central tendency (e.g. means) or other basic estimates (e.g. regression coefficient) AND variation (e.g. standard deviation) or associated estimates of uncertainty (e.g. confidence intervals)
☐	☒	For null hypothesis testing, the test statistic (e.g. *F*, *t*, *r*) with confidence intervals, effect sizes, degrees of freedom and *P* value noted<br>*Give P values as exact values whenever suitable.*
☐	☒	For Bayesian analysis, information on the choice of priors and Markov chain Monte Carlo settings
☒	☐	For hierarchical and complex designs, identification of the appropriate level for tests and full reporting of outcomes
☒	☐	Estimates of effect sizes (e.g. Cohen's *d*, Pearson's *r*), indicating how they were calculated

Our web collection on statistics for biologists contains articles on many of the points above.

Software and code

Policy information about availability of computer code

Data collection	MyGo Pro PCR Instrument (IT-IS Life Sciences, Ireland)<br>MyGo PCR Software 3.5.21 (IT-IS Life Sciences, Ireland)<br>GloMax 96 Luminumeter (Promega,USA)<br>R9.4.1 Flow Cell (Oxford Nanopore, UK)<br>Guppy (V4.4.2, V5.0.7) (Oxford Nanopore, UK)
Data analysis	Guppy (V4.4.2, V5.0.7) (Oxford Nanopore, UK)<br>Ubuntu Linux 18.04<br>BBDuk (v. 38.84)<br>MiniMap (v. 2.17)<br>Geneious Prime (v.2021.1.1.)<br>MAFFT webserver (v7)<br>IQ-Tree webserver (v1.6.8)<br>Beast (v.1.10.4)

For manuscripts utilizing custom algorithms or software that are central to the research but not yet described in published literature, software must be made available to editors and reviewers. We strongly encourage code deposition in a community repository (e.g. GitHub). See the Nature Portfolio guidelines for submitting code & software for further information.

Data

Policy information about availability of data

All manuscripts must include a data availability statement. This statement should provide the following information, where applicable:

- Accession codes, unique identifiers, or web links for publicly available datasets
- A description of any restrictions on data availability
- For clinical datasets or third party data, please ensure that the statement adheres to our policy

The genomic sequence data in this study have been deposited in the NCBI GenBank database under accession codes: MW775010https://www.ncbi.nlm.nih.gov/nuccore/MW775010, MW775011https://www.ncbi.nlm.nih.gov/nuccore/MW775011 and MZ541881 https://www.ncbi.nlm.nih.gov/nuccore/MZ541881. Tick samples: OL795929-OL795963 https://www.ncbi.nlm.nih.gov/nuccore/?term=ixodes+lloviu+hungary. The neutralization data generated in this study are provided in the Source Data file. The background data of sampled animals is provided in the Supplementary Data. Sequencing protocol and materials are listed here: https://www.protocols.io/view/lloviu-cuevavirus-sequencing-protocol-bmz3k78n.html. Source data are provided with this paper.

Field-specific reporting

Please select the one below that is the best fit for your research. If you are not sure, read the appropriate sections before making your selection.

- [x] Life sciences
- [ ] Behavioural & social sciences
- [ ] Ecological, evolutionary & environmental sciences

For a reference copy of the document with all sections, see nature.com/documents/nr-reporting-summary-flat.pdf

Life sciences study design

All studies must disclose on these points even when the disclosure is negative.

Sample size	No statistical test was used to determine sample size. Sample sizes were variable between sampling events, depending on the actual colony size and the availability of animals. During in vitro laboratory experiments, the LLOV positive sample number limited the number of experiment replicates.
Data exclusions	No data were excluded from the study
Replication	Depending on the available amount of bat sera different measurement and dilution strategies were applied: measured as duplicate with repeated experiment - bat1,169,170,99,115,130; measured in duplicate with single experiment – bat2,102,138; measured in single in one experiment – bat98,110,118,143. All dilutions were 1/40, 1/80, 1/160, 1/320, 1/640, 1/1280, 1/2560, 1/5120 except for bat143 where 1/100 starting dilution was applied.<br>In vitro isolation experiments were performed in triplicates with same results. The RNA FISH analysis was performed once. The probes in the RNA FISH experiment were evaluated with recombinant LLOV and reliably detected positive and negative sense recombinant LLOV RNA in infected cells (see Hume AJ, Heiden B, Olejnik J, Suder EL, Ross S, et al. (2022) Recombinant Lloviu virus as a tool to study viral replication and host responses. PLOS Pathogens 18(2): e1010268.).<br>All attempts of replications were successful.
Randomization	Randomization is not relevant in our study, since we sampled wild animals. This sampling is affected by multiple random factors, there is no option for randomized sampling. Animal activity, number of animals for sampling and accessibility of animals are not predictable. We sampled all animals which could be catched.
Blinding	Blinding is not relevant in our study, since we sampled wild animals and performed screening on site. This sampling is affected by multiple random factors, there is no<br>option for blinded sampling. Animal activity, number of animals for sampling and accessibility of animals are not predictable. Circumstances are not applicable for blinded sampling. Study design and relevance do not requires blinded sampling.

Reporting for specific materials, systems and methods

We require information from authors about some types of materials, experimental systems and methods used in many studies. Here, indicate whether each material, system or method listed is relevant to your study. If you are not sure if a list item applies to your research, read the appropriate section before selecting a response.

Materials & experimental systems

n/a	Involved in the study
☐	☒ Antibodies
☐	☒ Eukaryotic cell lines
☒	☐ Palaeontology and archaeology
☐	☒ Animals and other organisms
☒	☐ Human research participants
☒	☐ Clinical data
☒	☐ Dual use research of concern

Methods

n/a	Involved in the study
☒	☐ ChIP-seq
☒	☐ Flow cytometry
☒	☐ MRI-based neuroimaging

Antibodies

Antibodies used

EBOV convalescent serum (NIBSC WHO standard 15/262; 15/282) (NHSBT)
polyclonal antibody samples, these are the samples of wild animals collected during the study

Validation

https://www.nibsc.org/documents/ifu/15-262.pdf
https://www.nibsc.org/documents/ifu/16-344.pdf

Eukaryotic cell lines

Policy information about cell lines

Cell line source(s)

Cercopithecus aethiops (African green monkey kidney cells (VeroE6) ATCC CRL-1586; Miniopterus sp. kidney cell line SV40 transformed cell line (SuBK12-08, kindly provided by prof. Ayato Takada, Hokkaido University, Japan); human neuroblastoma cell line (SH-SY5Y, ATCC CRL-2266); human hepatocellular carcinoma cell line (Hep-G2, ATCC HB-8065); human non small-cell lung carcinoma (HCC 78, DSMZ ACC 563); human colon carcinoma (HCT 116, DSMZ ACC 581); Tadarida brasiliensis lung cell line (Tb-1 Lu, ATCC CCL 88); human kidney cell line (HEK 293T/1, ATCC CRL-11268); human kidney cell line (HEK 293T, ATCC CRL-3216)

Authentication

None of the cell used were authenticed.

Mycoplasma contamination

All cells were tested negative with a commercially available Mycoplasma detection PCR test.

Commonly misidentified lines
(See ICLAC register)

No commonly misidentified cell lines were used.

Animals and other organisms

Policy information about studies involving animals; ARRIVE guidelines recommended for reporting animal research

Laboratory animals

No laboratory animals were used in the study

Wild animals

Miniopterus schreibersii and Myotis myotis bat individuals were collected by lincenced chiropterologists. Animals were sampled on site and released after sampling and/or real-time RT-PCR testing into the same roost place where they were captured. All related animal information (including sex and approximate age) is included as a Supplemetary Data file.

Field-collected samples

Samples (blood, urine, feces, ectoparasites) were collected from Schreiber's bats (Miniopterus schreibersii) and Greater mouse-eared bats (Myotis myotis). Bat species identification was performed by trained chiropterologists according to morphological identification keys54. Considering conservational aspects, all sampling activities were conducted after the reproduction period of the colony (between August to November). During the live sampling events, serum samples were taken from captured bats after which they were left hanging separately in disposable paper bags (air permeable) for approximately 2hours, while on-site RT-PCR analysis for the presence of LLOV RNA in from blood samples was performed (as detailed in the Virus detection section). This methodology permitted the observation and re-sampling of infected bats. Altogether, 376 bat individuals were sampled between 2016 and 2020 (Supplementary Table). Whole blood (maximum of 50 µL) was taken from the uropatagium vein from each animal by Minivette® POCT (Sarstedt, Germany) disposable microtubes. At the first live animal sampling event (18.09.2018), the blood samples were collected in 1.5-ml Eppendorf tubes, where serum was separated for neutralization assays by low-speed centrifugation (1,000 g) for five minutes. Cell pellets were used for nucleic acid extraction and RT-PCR detection of LLOV RNA. Due to the multipurpose nature of the investigation and the strong limitation of the blood amount, in case of samples less than 8 µL, only LLOV RNA detection was conducted. When more blood was collected (approximately 8-13 µL), only serology was performed. For samples with volumes above 13 µL, both LLOV RT-PCR and serology testing were performed. Cold chain of the samples were maintained from the site to the laboratory with CX-100 dry shippers (liquid nitrogen). Sampling was performed under different temperature and weather conditions during daytime, under the actual seasonal characteristics.

Ethics oversight

Bat sampling activities on a country-wide scale were approved by the Hungarian Government Office of Pest County under the registration number of PE-KTFO/4384-24/2018. Animal handling was performed by licensed chiropterologists, no animals were harmed during the study, and all ethical standards were followed during the work.

Note that full information on the approval of the study protocol must also be provided in the manuscript.
